# Use of Clinical Disease Activity Index Score for Assessment of Disease Activity in Rheumatoid Arthritis Patients: An Indian Experience

**DOI:** 10.1155/2011/146398

**Published:** 2011-12-29

**Authors:** H. Singh, H. Kumar, R. Handa, P. Talapatra, S. Ray, V. Gupta

**Affiliations:** Department of Medicine, Pt. BDS PGIMS, Haryana Rohtak 124001, India

## Abstract

*Introduction*. Serial objective assessment of disease activity in Rheumatoid Arthritis (RA) is imperative to achieve remission. The CDAI score appears more practical than DAS-28 in routine assessment of disease activity in RA patients. *Objective*. To evaluate correlation and agreement of the DAS-28 with CDAI in RA patients. *Methods*. A total of 200 patients of RA were evaluated by DAS-28 and CDAI and divided into 4 categories of disease activity i.e. Group-I: Remission (DAS-28 < 2.6; CDAI < 2.8), Group II: Low disease activity (DAS-28 = 2.6–3.2; CDAI = 2.8–10), Group III: Moderate disease activity (DAS-28 = 3.2– 5.1; CDAI = 10–22), Group IV: High disease activity (DAS-28 > 5.1; CDAI > 22). DAS-28 was compared to CDAI in each group using spearman correlation coefficient and kappa statistics. *Results*. Group I shows mean DAS-28 of 1.99 ± 0.38; mean CDAI of 0.90 ± 0.65, (*P* = 0.0001). Group II shows mean DAS-28 of 3.04 ± 0.17; mean CDAI of 6.45 ± 02.35, (*P* = 0.0001). Group III shows mean DAS-28 of 4.25 ± 0.58; mean CDAI of 16.46 ± 3.31 (*P* < 0.0001). Group IV shows mean DAS-28 of 6.38 ± 0.87; mean CDAI of 38.56 ± 11.88 (*P* < 0.0001). Kappa statistics (*κ*) of the above comparison was 0.533. *Conclusion*. Our findings indicate that CDAI—a composite score that employs only clinical variables and omits assessment of Acute Phase Reactant (APR), has moderate to good correlation (Kappa value = 0.533) to DAS-28 for assessment of disease activity in RA patients.

## 1. Introduction

The diagnosis of patients with established Rheumatoid Arthritis (RA) is based upon symmetrical polyarthritis characteristically involving small joints of the hands with/without deformities. It is also established that duration of active disease is associated with joint damage and disability. Therefore, early initiation of treatment is needed to reduce structural damage in RA. The current treatment approach for patients with rheumatoid arthritis involves early initiation of aggressive therapy with disease-modifying drugs (DMARDS) and biologic agents. The goal of treatment is remission. Therefore, regular assessment of disease activity is necessary in the clinic for guiding the treatment. In this respect, the patients should understand the term “disease activity” as they understand their glucose values or blood pressure values in diabetes or hypertension, respectively. This can be the key for success of and compliance to the therapy. Since many years, the measurement of disease activity in RA has been done by Disease Activity Score-28 (i.e., DAS-28). DAS-28 is measured by assessing 28-tender-joint count (range 0–28), 28-swollen-joint count (range 0–28), Erythrocyte Sedimentation Rate (ESR) and general health on VAS scale (0–100 mm) [1]. DAS-28 is a continuous index ranging from 0 to 9.4, in which low disease activity as is defined as ≤3.2; moderate disease activity is defined as >3.2 to ≤5.1; high disease activity is defined as >5.1 [2]. A commonly used cutoff point for remission in DAS-28 is <2.6 [3].

 A newer tool for evaluation of disease activity is Clinical Disease Activity Index (CDAI). It is the only disease activity index without an acute-phase reactant [4].

The development of CDAI was originally based on the notion that the available nondichotomous disease activity index, that is, DAS-28, although ingenious as continuous scale and highly valuable in clinical studies in RA, might be too complicated for disease activity assessment in routine clinical practice. The complex formula of DAS-28 requires additional tools such as nomogram, a calculator, or a computer. The idea to simply employ a numerical summation of the values of derived set of disease activity variables reflecting inflammatory joint disease was first proven to be valid and sensitive to change in patients with reactive arthritis in the context of the development and validation of disease activity index for reactive arthritis (DAREA). Subsequently, this concept was implemented and validated for RA using several clinical trial datasets [5–8].

Clinical Disease Activity Index (CDAI) is a composite index (without acute-phase reactant) for assessing disease activity. CDAI is based on the simple summation of the count of swollen/tender joint count of 28 joints along with patient and physician global assessment on VAS (0–10 cm) Scale for estimating disease activity [4]. The CDAI has range from 0 to 76. Validity of CDAI was determined by Aletaha et al. by studying its correlational validity (refers to comparison with other measures of disease activity), discriminant validity (in this setting relates to the correlation of changes in the scale with changes in other measures of disease activity), and construct validity (considers correlation with important outcomes of the disease, such as radiological progression) by various statistical methods [9]. CDAI will prove greatest value in clinical practice rather than research, where acute-phase reactants are nearly always available. The greater advantage associated with CDAI is its potential to be employed in evaluation of patients with RA consistently with close frequency and independently of any calculating device, therefore, it can essentially be used everywhere and anytime for disease activity assessment in RA patients.

Also, CDAI cutoff values for remission are more stringent than DAS-28, since CDAI allows for lesser residual disease activity because DAS-28 < 2.4 allows up to 8 tender/swollen joint count while CDAI < 2.8 allows only less than 2 tender/swollen joint count [10]. Therefore, target for CDAI-categorized remission should be more beneficial to the patients in the terms of symptom control and disease morbidity.

 There is currently an effort to revise the definition of remission and low disease activity (both combined as Minimal Disease Activity state). In the past few years various studies have proposed different cutoff values for CDAI and also for DAS-28 (especially for remission group) [3, 4, 11]. To avoid any confusion, cutoff values of DAS-28 and CDAI as proposed by American College of Rheumatology-2008 (ACR-2008) criteria are used in present study.

Based on the above discussion, it was worthwhile to calculate the CDAI in Indian RA patients and assess its correlation to DAS-28, as there is a limited experience with CDAI in Indian setting.

So, the aim of the present study was to assess disease activity in RA patients using CDAI and to evaluate the correlation and agreement of CDAI with DAS-28.

## 2. Patients and Methods

The present study, which was a cross-sectional study, was conducted at Pt. BDS PGIMS, Rohtak to study evaluation of disease activity in Rheumatoid Arthritis patients using Clinical Disease Activity Index (CDAI). A total of 200 patients of RA (diagnosed as per American College of Rheumatology 1987 revised criteria) were included as subjects in the study. Those patients who were suffering from severe anemia, hypothyroid, having renal, cardiac, liver, or pulmonary disease were excluded from the study group. All the selected patients satisfied the inclusion and exclusion criteria. Written consent was taken from all patients being subjects in the study. All the subjects included in the study were detailed for their history, clinical examination, and routine laboratory investigations including radiographic examination. 

At each visit, clinical, functional, and laboratory parameters and disease activity core set variables according to the composite scores DAS-28 and CDAI were documented. 

All subjects were scored for 28-joint count (tender/swollen), Global Health assessment (GH) using Visual Analog Scale (VAS in 0 to 100 mm), Patient's Disease Global Assessment (PDGA) as per VAS (0 to 10 cm), Evaluator Disease Global Assessment (EDGA) using VAS (0 to 10 cm), and ESR (using Westergren's method, in mm/first hour). Independent, trained assessors who were not involved in treatment decisions performed clinical assessments including joint counts. 

With the use of above measurements DAS-28 was calculated by using the following formula:

(1) $$\begin{array}{cc} & \text{DAS-}28\\ & \;=0.56\;\sqrt{\text{TJC}}+0.28\;\sqrt{\text{SJC}}+0.70\;\left(\text{ln ESR}\right)+0.014\;\left(\text{GH}\right),\; \end{array}$$

where TJC is the Tender Joint Count, SJC is the Swollen Joint Count, ESR is the Erythrocyte Sedimentation Rate, and GH is the Global Health on VAS Scale (0–100 mm). 

 Also, Clinical Disease Activity Index (CDAI) of the patients at the same visit was performed by the following formula:

(2) CDAI=TJC+SJC+PDGA+EDGA,

where TJC is the Tender Joint Count, SJC is the Swollen Joint Count, PDGA is the Patient's Disease Global Assessment (VAS 0–10 cm), and EDGA is the Evaluator/Assessor's Disease Global Assessment (VAS 0–10 cm). 

For the purpose of statistical calculations DAS28 and CDAI values were taken on each patient. DAS-28 and CDAI thus calculated were subjected to statistical analysis for the predictability of CDAI in disease activity of Rheumatoid Arthritis. Correlational validity of CDAI with DAS-28 was assessed using Spearman rank correlations and kappa (*κ*) statistics. 

## 3. Results 

All 200 patients were grouped in four categories (Group I: remission; Group II: low disease activity; Group III: moderate disease activity; Group IV: high disease activity) according to the values based upon DAS-28 and CDAI separately (as per ACR-2008 guidelines) (Tables 1 and 2). Of the 200 subjects there were 161 females and 39 males (M : F ratio = 1 : 4.13); the mean age for the study group was 41.82 ± 12.78 years, and the mean duration of illness in the study group was 5.49 ± 5.34 years (Table 3). The results in present study show that median TJC was 9 (quartile range 5–18), median SJC was 4 (quartile range 2; 6), median PGA was 5 (quartile range 2; 7), median EGA was 3 (quartile range 1.5; 3), and median ESR was 40 (quartile range 30; 50) (Table 4). 

All 200 patients were divided into 4 groups of disease activity based on DAS-28 as mentioned earlier: there were 9 patients in Group I, 5 patients in Group II, 62 patients in Group III, and 124 patients in Group IV (Table 1). Further, these patients were grouped into four categories of disease activity based on CDAI: there were 11 patients in Group I, 31 patients in Group II, 62 patients in Group III, and 96 patients in Group IV (Table 2). The mean DAS-28 group of the study Group was 5.5 ± 1.49, and the mean CDAI was 25 ± 16.35. The CDAI was compared to DAS-28 of total 200 patients using Spearman's correlation coefficient method and kappa score cross-tabulation method (total 200 patients and individual groupwise comparison; Table 5). When mean CDAI was compared to mean DAS-28 of the whole group, Spearman correlation coefficient was 0.980 with *P*  value ≤ 0.0001. (Table 5). For remission group (Group I), Sprearman's correlation coefficient was 0.975 with *P*  value ≤ 0.0001 (VHS). For low disease activity group (Group II), Sprearman's correlation coefficient was 0.908 with *P*  value = 0.001. For moderate disease activity group (Group III), Sprearman's correlation coefficient was 0.997 with *P*  value ≤ 0.0001 (VHS); for high disease activity group (Group IV), Sprearman's correlation coefficient was 0.999 with *P*  value ≤ 0.0001 (VHS) (Table 5). Measurement of agreement between CDAI and DAS-28 using Kappa value (*κ* = 0.533) was moderately good. 

## 4. Discussion

Therapy for rheumatoid arthritis has seen great progress over the past 10 years, including the approval of new drugs and the implementation of new strategies. Given these possibilities, long-term remission, normalization of physical function, and sustained quality of life are now achievable for many patients. In the western countries, the use of objective disease activity measures is commonly employed in the clinical setting for the care of individual patients. However, in India clinicians have been more reluctant to agree upon the routine use of an objective disease measure (based on either patient-reported or physician-measured outcomes) [12]. 

Demonstration that routine measurement of disease activity in clinical practice correlates with improved patient outcomes (independent of aggressive disease control) would be the most compelling evidence that disease activity measures should be routinely measured. The commonly used disease activity measurement tool in RA is DAS-28. With DAS-28 some limitations have emerged, as they tend to do with any measure, for example, the lower specificity of the DAS-28 when it comes to low disease activity and in particular remission, not to mention its complex formula. 

Aletaha et al. originally devised CDAI from Simplified Disease Activity index (SDAI) in an attempt to prove the insignificant contribution of acute-phase reactants (CRP and ESR) to DAS-28 and SDAI [13]. CDAI is a simple summation score requiring nothing more complex than addition [14]. The merit of CDAI is very obvious given its sheer simplicity for usage in clinical practice without the need of any calculating device or laboratory parameter [4]. In the Indian context, where a large percentage of patients may not be carrying their ESR or CRP values due a multitude of reasons, CDAI is probably the best instrument that any physician can use on a day-to-day basis for assessing disease activity in RA. So, the present study was planned to evaluate disease activity in RA patients using CDAI and to compare the correlation and concordance of CDAI with respect to DAS-28. 

Mean DAS-28 of our study group was 5.5 ± 1.49 (mean ± SD), and mean CDAI of the study group was 25 ± 16.35. Spearman's correlation coefficient (*r*) was 0.980 with *P* value of < 0.0001 (Table 5). In the Indian study by Arya et al., DAS-28 was 5.97 ± 1.21 and mean CDAI of the study group 32.62 ± 15.49 [15]. 

In the present study 200 RA patients were first classified according to CDAI cutoff values (Tables 1, 2, and 5) and cross tabulation chart of CDAI and DAS-28 made (Table 5) using kappa statistics, and kappa score was calculated for CDAI versus DAS-28 to be 0.533 (*P* < 0.0001) proving moderately good correlation. The original study by Aletaha et al. in 2005 showed a kappa score of 0.70 between CDAI and DAS-28 (*P* < 0.0001) [14]. A study by Greenberg et al., in 2009 showed a moderate-to-substantial agreement between CDAI and DAS-28 (EULAR) with kappa score = 0.58 (*P* < 0.0001) [16]. Günaydin et al. showed a good agreement (kappa = 0.705 to 0.765) between DAS-28 and CDAI at different levels of disease activity [17]. In the study by Rintelen et al., the agreement between DAS-28 categories and CDAI categories was moderate with kappa = 0.525 (*P* < 0.0001) [18]. Also, Ranganath et al. showed 78–80% agreement between CDAI and DAS-28 for various categories of disease activity [19]. These all studies showed nearly similar kappa values and level of significance as in present study. 

Comparison of DAS-28 to the CDAI was made using Spearman's correlation analysis (Table 5). Each group of disease activity (as per DAS-28) was compared to same group of disease activity (as per CDAI) (Table 5). The above analysis showed spearman's coefficient (*r*) = 0.975 (*P* < 0.0001; VHS) for Group I (remission);  *r* = 0.908 (*P* = 0.001; VHS) for Group II (low disease activity);  *r* = 0.997 (*P* < 0.0001; VHS) for Group III (moderate disease activity);  *r* = 0.999 (*P* < 0.0001; VHS) for Group IV (high disease activity), (Table 5). The study by Rintelen et al., showed that kappa statistics revealed a moderate degree of agreement (*κ* = 0.525;  *P* < 0.0001) with respect to mild (Group II), moderate (Group III) and of high disease activity (Group IV) according to CDAI and DAS-28 with the exception concerning the definition of remission (Group I) [18]. Similarly, in a study by Shaver et al., there was only a fair agreement (*κ* = 0.2–0.4) for Minimal Disease Activity (includes both low disease activity and remission) between CDAI and DAS-28 [20]. 

The major limitation of this study was that it was not aimed to validate the cutoff values of CDAI, so it is possible only to correlate the CDAI to DAS-28 but not to improve already proposed CDAI cutoff values. Also, unexpectedly there was slightly more number of patients remission in CDAI group than DAS-28 group (9 patients in remission as per DAS-28 and 11 patients in remission as per CDAI), therefore larger prospective studies are required to validate the CDAI cutoff values for remission group. 

Our findings indicate that the CDAI—a composite score that employs only clinical variables and omits assessment of an APR—has moderately good correlation with DAS-28 for assessment of disease activity in patients with RA (*κ* = 0.533;  *P* < 0.0001). Also, because of simple numerical summation, CDAI is very easy to calculate. For these reasons, the CDAI should facilitate decision-making by physicians and helps to avoid lags in efficient treatment adaptation for RA patients. According to current knowledge, such intensified and prompt patient care can be expected from physicians to reduce the individual and socioeconomic impact of the disease in the longer term. 

## 5. Conclusion

In the present study we showed that the CDAI, a simple composite index obtained by numerical summation of four solely clinical variables, has a moderate-to-good correlation (*κ* = 0.533;  *P* < 0.0001) with DAS-28 for disease activity assessment in RA patients. On the basis of the study results and related statistics, it is suggested that CDAI has good concordance with DAS-28 for disease activity assessment in Rheumatoid Arthritis patients. Also, CDAI is easy to use in day-to-day clinical practice without the need of any lab value or any calculator/computer device. Therefore, CDAI is a very useful disease activity assessment tool in daily clinical practice for RA patients. 

## Figures and Tables

**Table 1:** Showing patients grouped according to DAS-28 cutoff values for staging of disease activity (ACR-2008).

Groups	Level of disease activity	DAS-28 (total score = 0–9.4)	Number of patients	Mean DAS-28 ± SD
I	Remission	<2.6	9	1.99 ± 0.38
II	Low disease activity	≤3.2	5	3.04 ± 0.17
III	Moderate disease activity	>3.2 and ≤5.1	62	4.25 ± 0.58
IV	High disease activity	>5.1	124	6.38 ± 0.87

Total		0–9.4	200	5.5 ± 1.49

**Table 2:** Showing patients groups according to CDAI cutoff values of different stages of disease activity.

Groups	Level of disease activity	CDAI	Number of patients	Mean CDAI-Score ± SD
I	Remission	<2.8	11	0.90 ± 0.65
II	Low disease activity	≤10	31	6.45 ± 2.35
III	Moderate disease activity	>10 and ≤22	62	16.46 ± 3.31
IV	High disease activity	>22	96	38.56 ± 11.88

Total		0–76	200	25 ± 16.35

**Table 3:** Showing demographic profile as per DAS-28.

Group	DAS-28	Duration of illness (Mean ± SD)	Mean Age ± SD	Sex	
				Number of males	Number of females
I	<2.6	1.56 ± 0.88	35.89 ± 8.50	1	8
II	≤3.2	6.40 ± 3.94	40.00 ± 15.64	1	4
III	>3.2 and ≤5.1	4.49 ± 4.24	39.70 ± 12.94	16	46
IV	>5.1	6.18 ± 5.88	42.70 ± 12.82	21	103

Total	0–9.4	5.49 ± 5.34	41.82 ± 12.78	39	161

**Table 4:** Showing median value of core set of variables.

Core set of variables	Median	Interquartile range (1st; 3rd quartile)
TJC	9	5–18
SJC	4	2; 6
PGA (0–10 cm)	5	2; 7
PGA (0–100 mm)	50	20; 70
EGA (0–10 cm)	3	1.5; 5
EGA (0–100 mm)	30	15; 50
ESR 0–200 mm)	40	30; 50

**Table 5:** Showing comparison of values between DAS-28 and CADI Score.

Comparison groups	Mean DAS-28 ± SD	Mean CDAI-ACR ± SD	Spearman's coefficient (*r*)	*P* value
I versus I	1.99 ± 0.38	0.90 ± 0.65	0.975	0.0001
II versus II	3.04 ± 0.17	6.45 ± 2.35	0.908	0.0001
III versus III	4.25 ± 0.58	16.46 ± 3.31	0.997	<0.0001
IV versus IV	6.38 ± 0.87	38.56 ± 11.88	0.999	<0.0001

Total	5.5 ± 1.49	25 ± 16.35	0.980	<0.0001

Kappa value of above comparison = 0.533 (moderate-to-good correlation:  *P* < 0.0001).
